# Research on the Welding Process and Weld Formation in Multiple Solid-Flux Cored Wires Arc Hybrid Welding Process for Q960E Ultrahigh-Strength Steel

**DOI:** 10.3390/ma17133178

**Published:** 2024-06-28

**Authors:** Ting Xiang, Mingrui Zhang, Qiang Ma, Zhenlong Fang, Huan Li, Hao Wang

**Affiliations:** 1 Tianjin Key Laboratory of High Speed Cutting and Precision Machining, Tianjin University of Technology and Education, Tianjin 300222, China; xiangting123@tju.edu.cn (T.X.); zmr1811836720@163.com (M.Z.);; 2 Technology Department of Tianjin Golden Bridge Welding Materials Group Co., Ltd., Tianjin 300300, China; 3 Tianjin Key Laboratory of Advanced Joining Technology, Tianjin University, Tianjin 300072, China

**Keywords:** ultrahigh-strength steel, multiple solid-flux cored wires arc hybrid welding, metal transfer, weld formation, mechanical properties

## Abstract

This paper proposes a novel welding process for ultrahigh-strength steel. The effects of welding parameters on the welding process and weld formation were studied to obtain the optimal parameter window. It was found that the metal transfer modes of solid wires were primarily determined by electrical parameters, while flux-cored wires consistently exhibited multiple droplets per pulse. The one droplet per pulse possessed better welding stability and weld formation, whereas the short-circuiting transfer or one droplet multiple pulses easily caused abnormal arc ignition that decreased welding stability, which could easily lead to a “sawtooth-shaped” weld formation or weld offset towards one side with more spatters. Thus, the electrical parameters corresponding to one droplet per pulse were identified as the optimal parameter window. Furthermore, the weld zone (WZ) was predominantly composed of AF, and the heat-affected zone (HAZ) primarily consisted of TM and LM. Consequently, the welded joint still exhibited excellent mechanical properties, particularly toughness, despite higher welding heat input. The average tensile strength reached 928 MPa, and the impact absorbed energy at −40 °C for the WZ and HAZ were 54 J and 126 J, respectively. In addition, the application of triple-wire welding for ultrahigh-strength steel (UHSS) demonstrated a significant enhancement in post-weld deposition rate, with increases of 106% and 38% compared to single-wire and twin-wire welding techniques, respectively. This process not only utilized flux-cored wire to enhance the mechanical properties of joints but also achieved high deposition rate welding.

## 1. Introduction

In recent years, countries have been actively promoting green and low-carbon industries [1]. High-strength steel (HSS) or ultrahigh-strength steel (UHSS) are extensively utilized as superior alternatives to traditional carbon steel in various sectors, including railways, ships, as well as engineering and mining machinery [2,3,4]. The utilization of high-strength structural steels not only enables lightweight constructions by reducing sheet thickness but also presents new possibilities to overcome current challenges in different technology fields [5,6]. The welding process plays a crucial role in manufacturing HSS or UHSS components and directly determines the quality and reliable operation of HSS and UHSS components, particularly for large-scale components [7]. However, it is difficult to ensure the toughness of the welded joint after welding, especially its low-impact toughness, which restricts the widespread adoption of UHSS at lower temperatures [8,9].

Weld alloying is an effective method to optimize both the toughness and strength of welded joints. As the quantity of acicular ferrite (AF) in the weld metal (WM) increases, the tensile strength and impact toughness of the welded joint also improve [10]. The addition of ferrite-forming elements such as V, Cr, Nb, and Ti can promote the formation of AF in the weld metal [11,12,13]. To achieve the desired morphology and properties of welded joints, extensive research has been conducted on optimizing the addition levels of alloying elements, as well as the welding process [14,15,16]. Indeed, the addition of alloying elements typically necessitates the utilization of consumable electrodes, such as solid wire or flux-cored wires. Compared to solid wire, flux-cored wire is filled with metal powder, making it easier to adjust the content of alloying elements inside [17]. This allows for precise control over the composition and properties of the weld [18]. Zhang et al. [19] investigated the microstructure and mechanical properties of the deposited metals from five metal-cored wires for welding 600–900 MPa ultralow carbon bainitic steels. Smolentsev et al. [20] found that using austenitic class flux-cored wire with nitrogen for welding high-strength alloy steels made it possible to obtain defect-free welded joints with an austenitic-ferritic structure. Wang et al. [21] found that the joint welded with an E81T1-Ni1M flux-cored wire was well formed without obvious welding defects, and the center of the weld was mainly needle-like ferrite. Mukhopadhyay et al. [22] found that acceptable weld metal properties in HSLA steel could be achieved with the proper combination of flux-cored wire and shielding gas composition. Ai et al. [11] analyzed the strengthening and toughening effects of Zr on weld metal in the welding process of Q960 steel. The result indicated that the highest AF content in the weld metal was observed when the weld metal contained 0.0061% Zr. The weld metal exhibited a certain amount of equiaxed fine-grained ferrite, with the lowest content of proeutectoid ferrite, which effectively improved the strength and toughness of the weld metal. Zou et al. [23] employed flux-cored wire for welding Q960 low-alloy high-strength steel and studied the effect of Al on the microstructure and mechanical properties of weld metal. The findings revealed that the Al addition could promote the formation of Al_2_O_3_ oxide inclusions, thereby promoting AF nucleation and improving the mechanical properties of weld metal. Bhole et al. [24] found that the increase in the Mo addition promoted the formation of a microstructure predominantly composed of ferrite in weld metal, and its toughness was significantly improved. When the Mo addition is 0.881 wt%, the weld metal has the optimal impact toughness at −45 °C.

As mentioned in the studies above, the majority of the welding processes for HSS and UHSS employ a single fluxed-cored wire. Despite the excellent joint performance achieved through flux-cored wire welding, there are limitations to further enhancing welding efficiency [25]. Therefore, this paper proposes a novel hybrid welding process for UHSS that could achieve superior weld quality and higher welding efficiency, i.e., the multiple solid-flux cored wires arc hybrid welding process. This process integrates three wires arranged in an equilateral triangle configuration into the same welding gun. Within this arrangement, the front-leading wires are two solid wires, while the following wire is flux-cored wire. By optimizing the alloying composition of the flux-cored wire, it is feasible to enhance the mechanical properties of the welded joint. Through the simultaneous arcing of the three wires during welding, a significant improvement in welding efficiency can be achieved. This process would unleash novel possibilities for high-quality and efficient welding of UHSS.

## 2. Materials and Methods

### 2.1. Welding Consumables and Equipment

**Figure 1:**
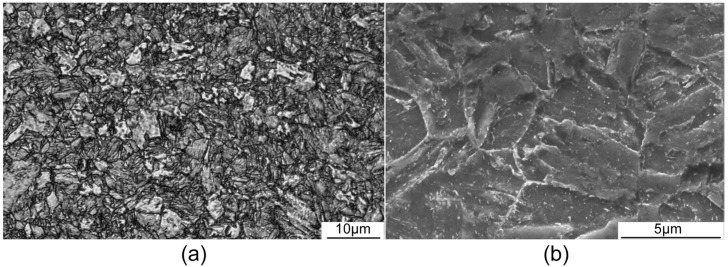
Initial microstructure of Q960E: (**a**) optical microscopy (OM) and (**b**) scanning electron microscopy (SEM).

The base metal employed was Q960E (Hunan Valin Xiangtan Iron and Steel Co., Ltd., Hunan, China), a low-alloy, high-strength steel after quenching and tempering heat treatment, with dimensions of 200 mm × 150 mm × 14 mm. The quenching process involved heating the material to a temperature of 900 °C and holding it for 10 min for water quenching, followed by high-temperature tempering at 630 °C for 15 min, and the heat treatment atmosphere was N_2_ to prevent oxidation. The chemical compositions of the welding consumables are presented in Table 1. The mechanical properties of the base metal are shown in Table 2. The base metal was observed using a Zeiss-AxioVert.A1 optical microscope (OM) (Carl Zeiss AG, Oberkochen, Germany), and a Sec-ALPHA scanning electron microscope (SEM, Shanghai SEC Inspection Equipment Co., Ltd., shanghai, China) at an operating voltage of 20 kV. The initial microstructure was tempered martensite (TM), which refers to spherical carbide particles dispersed on a ferrite matrix, as shown in Figure 1a,b. Furthermore, the primary constituents dispersed on the ferrite matrix are alloy M_3_C or M_7_C_3_-type carbides [26,27]. The filler wires consisted of two solid wires (JQ.MG90-G, Technology Department of Tianjin Golden Bridge Welding Materials Group Co., Ltd., Tianjin, China) and one flux-cored wire (JQ.YJ80ML, Technology Department of Tianjin Golden Bridge Welding Materials Group Co., Ltd., Tianjin, China), all with diameters of 1.2 mm. The experiment employed shielding gas composed of 82% Ar and 18% CO_2_, with a gas flow rate of 20 L/min. This study involved two welding forms: cladding and butt welding and the welding speed was set at 8 mm/s.

**Figure 2:**
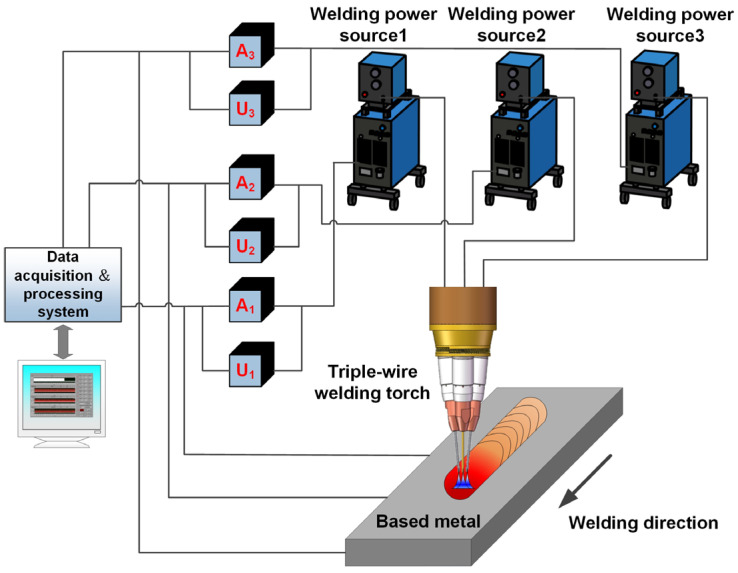
Schematic diagram of the welding system.

**Figure 3:**
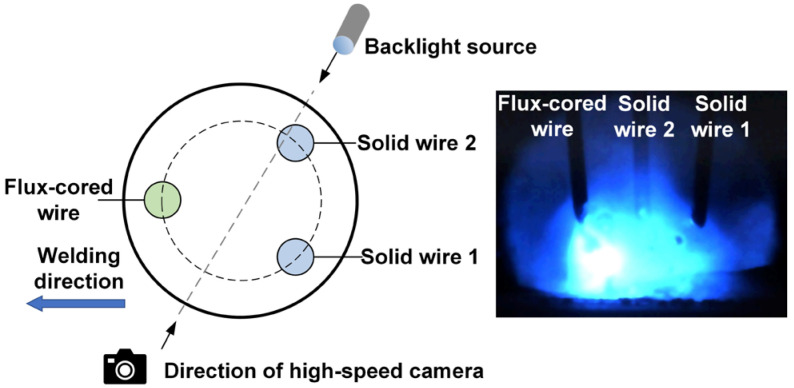
Corresponding relationship between the three-wire arrangement and the positions in high-speed photograph.

The welding equipment mainly consisted of the welding system and data acquisition system, as shown in Figure 2. The welding system primarily included three pulse welding power sources, a specially designed triple-wire welding torch, and a welding travel mechanism. The welding torch mainly consisted of three mutually insulated wires arranged in an equilateral triangle distribution, and each wire was powered by a separate welding power source. The flux-cored wire was positioned in the front as a guide during the welding process, while the two solid wires followed behind. The data acquisition system mainly consisted of an electrical signal acquisition system and a high-speed photography system. The electrical signal acquisition system monitored changes in electrical signals during the welding process, such as pulse current and voltage. The high-speed photography system monitored the arc behavior and metal transfer process. The corresponding relationship between the arrangement of the three wires and their positions in the high-speed photograph is illustrated in Figure 3. Starting from right to left, the first wire was the flux-cored wire, corresponding to the electrical signals *U*_FCW_ and *I*_FCW_. The second wire was solid wire 2, corresponding to the electrical signals *U*_SW2_ and *I*_SW2_. The third wire was solid wire 1, corresponding to the electrical signals *U*_SW1_ and *I*_SW1_.

### 2.2. Metallographic Analysis and Mechanical Property Test

**Figure 4:**
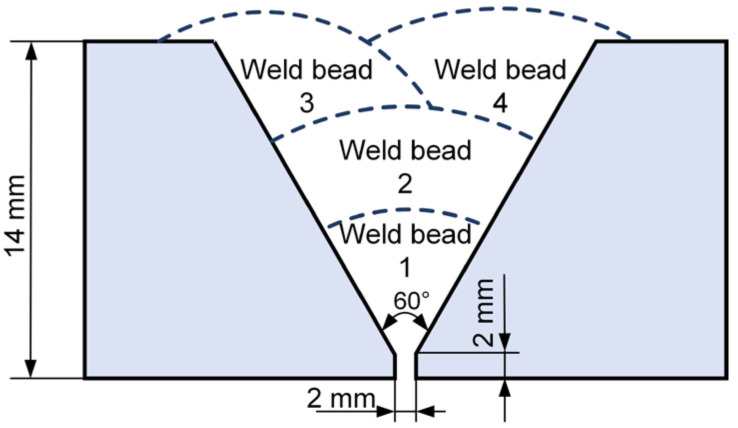
Groove dimensions of butt welding.

**Figure 5:**
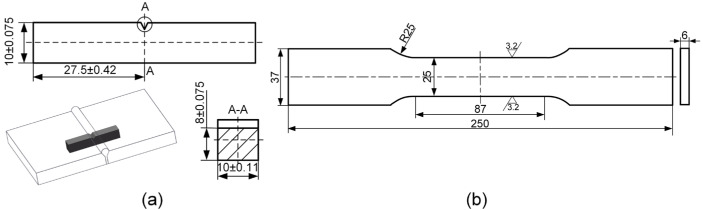
Dimensions of specimens for mechanical property test: (**a**) impact specimen and (**b**) tensile specimen.

The workpieces for butt welding were machined into a Y-groove with an angle of 60° and a root face thickness of 2 mm, and a root gap of 2 mm was kept between two workpieces, as shown in Figure 4. After butt welding, specimens were cut perpendicular to the welding direction from the welded joint. These specimens were ground, polished, and then etched with a 4% HNO_3_ alcohol solution to observe the microstructures by OM and SEM. In addition, tensile tests were conducted using a 30-ton MTS universal testing machine (MTS Systems Corporation, Minnesota, USA) at room temperature, with a loading rate of 1.2 mm/min, and tensile samples were machined according to the GB/T2651-2023 [28] standard, as shown in Figure 5b. Charpy V-notch testing was conducted at −40 °C using samples machined in accordance with the GB/T229-2020 [29] standard, as shown in Figure 5a. The V-notch, with an angle of 60°, was machined in accordance with the GB/T2650-2022 [30] standard both in the weld and the heat-affected zone.

## 3. Results and Discussion

### 3.1. Effects of Electrical Parameters on Metal Transfer and Arc Behavior

The optimization of welding parameters plays a pivotal role in achieving a stable welding process and a well-formed weld bead. Therefore, the analysis focuses on investigating the effects of different preset currents and voltages on arc behavior and metal transfer.

#### 3.1.1. Effects of Preset Voltage on Metal Transfer and Arc Behavior

The experiment employed a preset current of 140 A, corresponding to respective preset voltages of 22 V, 26 V, and 30 V, to illustrate three types of metal transfer modes. The three wires were synchronized with a pulse phase difference of 0° to ensure a synchronous arcing state. Furthermore, all three welding power sources retained identical preset parameters.

**Figure 6:**
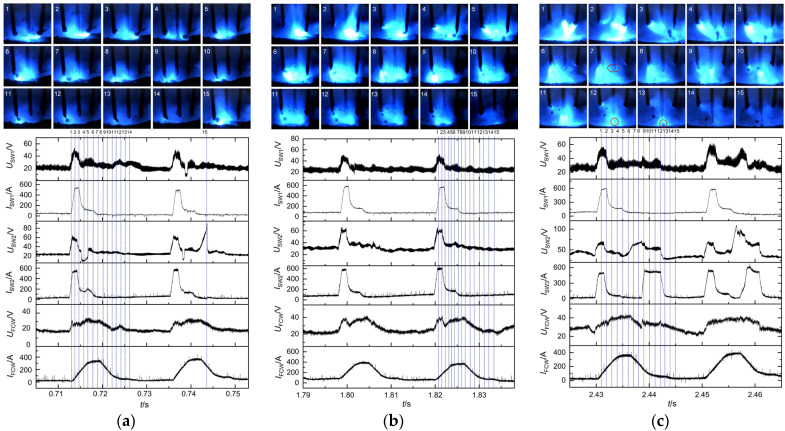
Electrical signals and high-speed photographs at different preset voltages: (**a**) preset voltage of 22 V, (**b**) preset voltage of 26 V, and (**c**) preset voltage of 30 V.

Electrical signals and high-speed photographs captured at the preset voltage of 22 V are depicted in Figure 6a. Initially, solid wires 1 and 2 rapidly ignited arcs as the pulse peak currents were applied (refer to images 1 and 2). Meanwhile, the flux-cored wire demonstrated an extended arcing period characterized by a delayed pulse peak current. Consequently, only a faint arc light was observed at the wire end. Subsequently, the ends of solid wires 1 and 2 formed droplets (refer to image 3). Due to the short arc length, a momentary contact between the droplet at the end of solid wire 2 and the weld pool resulted in short-circuiting transfer (refer to image 4), leading to an abrupt voltage drop. At this moment, the flux-cored wire began to burn the arc intensively as the pulse peak currents were applied. Then, the droplet at the end of the flux-cored wire detached and transferred to the weld pool (refer to images 7 and 8). Because the flux-cored wire, filled with metal powder, exhibited an extended arcing period, it was more likely to generate multiple droplets per pulse compared to the solid wires (refer to images 6–14). The solid wires exhibited a higher propensity to form short-circuiting transfer at lower preset voltage, which led to abnormal fluctuations in the pulse signal. For instance, solid wire 2 experienced an abrupt arc ignition (refer to image 15).

**Figure 7:**
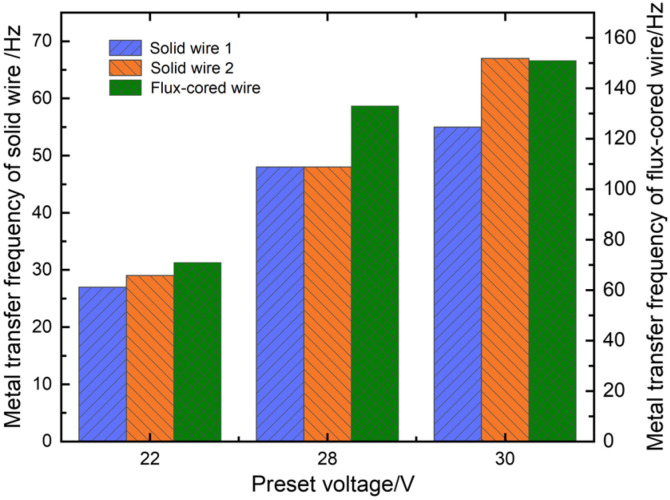
Effect of preset voltage on metal transfer frequency.

The arc lengths increased proportionally with the increase in arc voltage to 26 V, and short-circuiting transfer ceased to occur, as shown in Figure 6b. In addition, the electrical signals exhibited increased regularity, and the welding process kept smooth and stable metal transfers of one droplet per pulse for solid wires and multiple droplets per pulse for flux-cored wires. When the preset voltage increased to 30 V, solid wire 2 generated metal transfer of one droplet multiple pulses, as illustrated in Figure 6c. Initially, solid wire 1 and the flux-cored wire accomplished metal transfer during the first pulse period. However, a droplet consistently remained at the end of solid wire 2 (indicated by a red circle in image 7). The droplet did not detach from the end of solid wire 2 and transfer to the weld pool until the second pulse period (indicated by red circles in images 12 and 13). Furthermore, as the arc lengths significantly increased, the arc deflections towards each other became more pronounced, which led to more intense interference among arcs. Consequently, abnormal arc ignition frequently occurred and even caused the streaming transfer, such as solid wire 2. The abnormal arc ignition would reduce welding stability. As illustrated in Figure 7, the metal transfer frequencies of the three wires increased with the increase in preset voltage. This was because the pulse frequency increased, and short-circuiting transfer also disappeared as the arc length increased. Furthermore, the flux-cored wire had the highest transfer frequency due to its easy melting, followed by the solid wires.

#### 3.1.2. Effects of Preset Current on Metal Transfer and Arc Behavior

**Figure 8:**
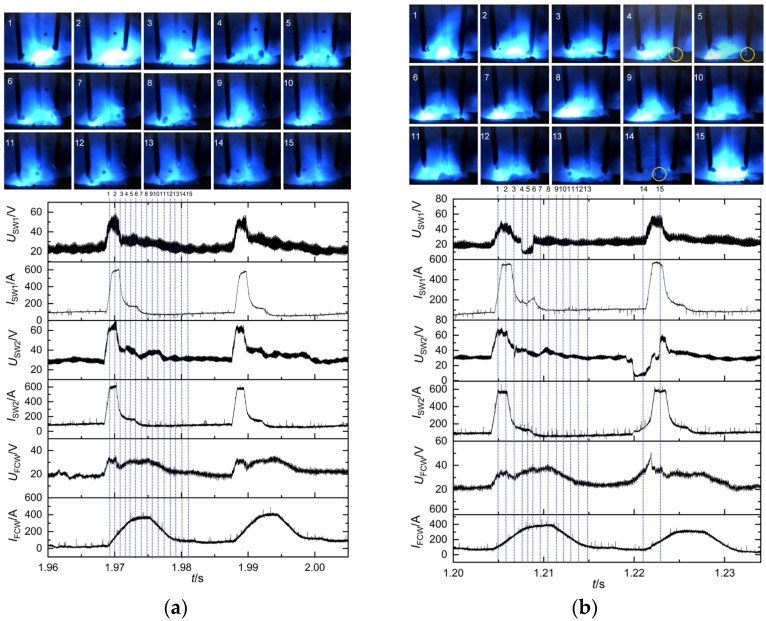
Electrical signals and high-speed photographs at different preset currents: (**a**) preset current of 160 A and (**b**) preset current of 180 A.

**Figure 9:**
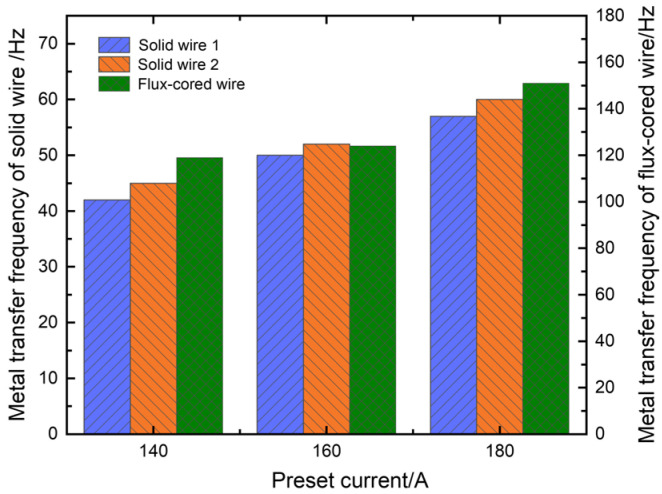
Effect of preset current on metal transfer frequency.

The experiment employed a preset voltage of 26 V, corresponding to respective preset currents of 140 A, 160 A, and 180 A. Three welding power sources kept the same preset parameters. The detailed metal transfer process at a preset current of 140 A is illustrated in Figure 6b. The whole welding process remained relatively stable at preset currents of 140 A and 160 A. Both solid wires 1 and 2 exhibited a metal transfer mode of one droplet per pulse, while the flux-cored wire demonstrated a metal transfer mode of multiple droplets per pulse. When the preset current increased to 180 A, short-circuiting transfer occurred at the end of solid wire 1, as indicated by the yellow circles in Figure 8b, which led to poorer welding stability. In addition, the metal transfer frequencies of the three wires exhibited an upward trend as the preset current increased, as illustrated in Figure 9. Welding current directly determined pulse frequency; therefore, an increase in preset current resulted in a significant increase in transfer frequency.

### 3.2. Arcing and Metal Transfer Modes of Multiple Solid-Flux Cored Wires Arc Hybrid Welding

**Figure 10:**
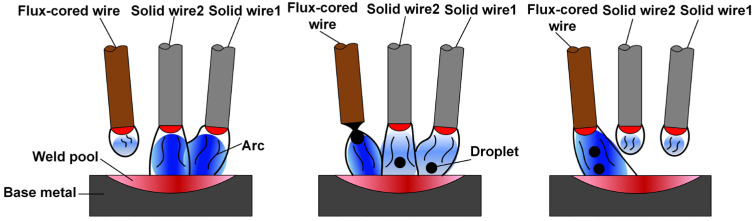
Arcing and metal transfer mode for multiple solid-flux cored wires arc hybrid welding.

The arcing and metal transfer modes of the multiple solid-flux cored wires arc hybrid welding are illustrated in Figure 10. It can be observed that solid wires 1 and 2 exhibited a higher priority in reaching the pulse peak phase compared to the flux-cored wire. Initially, droplets formed at the ends of solid wires 1 and 2 and then transferred to the weld pool, while the flux-cored wire only formed the droplet without detaching. Subsequently, under the action of electromagnetic force, the droplets detached from the wire ends and transferred into the weld pool. Due to the extended pulse peak period for the flux-cored wire, it consistently maintained multiple droplets per pulse during the welding process.

### 3.3. Effects of Electrical Parameters on Weld Formation

#### 3.3.1. Effect of Preset Voltage on Weld Formation

**Figure 11:**
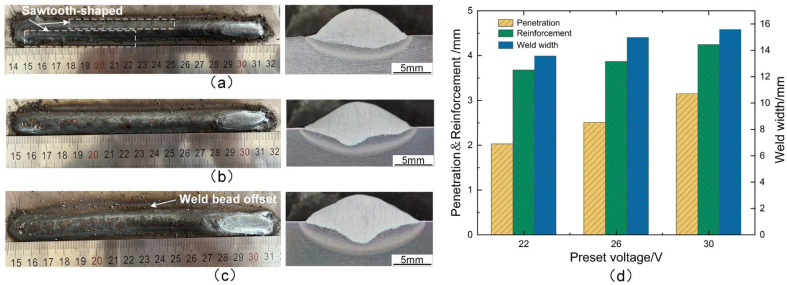
Effect of different preset voltages on weld formation: (**a**) preset voltage of 22 V, (**b**) preset voltage of 26 V, (**c**) preset voltage of 30 V, and (**d**) weld size.

The weld formations and cross-sectional morphologies at the preset voltages of 22 V, 26 V, and 30 V are illustrated in Figure 11a, Figure 11b, and Figure 11c, respectively. The weld edge exhibited insufficient smoothness at 22 V, as depicted in Figure 11a. Additionally, the weld width and penetration were relatively small, measuring merely 13.58 mm and 2.03 mm, respectively. This was primarily because the frequent short-circuiting transfer led to the pronounced vibrations in the weld pool at the lower preset voltage. As a result, the weld edges became uneven and even exhibited a “sawtooth” appearance. The weld formation exhibited a smooth and uniform appearance at 26 V, as depicted in Figure 11b. Moreover, both the weld width and penetration noticeably increased. From Figure 11c, it is evident that the weld bead had offset to one side, and there were more spatters. Additionally, both the weld width and penetration reached maximums of 3.15 mm and 15.59 mm, respectively. The higher the preset voltage increased, the greater the arc deflection became, which made it more likely for the weld bead to offset towards one side. Furthermore, the metal transfer followed the direction of arc deflection rather than vertical descent, resulting in larger splatters.

#### 3.3.2. Effect of Preset Current on Weld Formation

**Figure 12:**
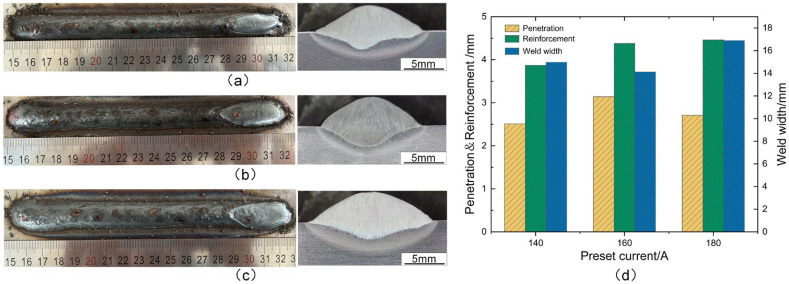
Effect of preset currents on weld formation: (**a**) preset current of 140 A, (**b**) preset current of 160 A, (**c**) preset current of 180 A, and (**d**) weld size.

The weld formations and cross-sectional morphologies corresponding to the preset currents of 140 A, 160 A, and 180 A are illustrated in Figure 12a, Figure 12b, and Figure 12c, respectively. It can be observed that all three weld beads were well-formed, with the minimum weld penetration measuring only 2.51 mm at 140 A and reaching a maximum of 3.14 mm as the current increased to 160 A. When the preset current continued to increase to 180 A, the weld width reached its maximum of 16.89 mm, while the weld penetration decreased to 2.71 mm. The increase in current led to a significant rise in the metal transfer frequency, thereby attaining the maximum deposition rate. Therefore, both the weld width and reinforcement reached their maximum values, as depicted in Figure 12c. However, with the preset current increased to 180 A, short-circuiting transfer occurred frequently. The higher occurrence of short-circuiting transfer weakened the impact of solid wire arcs on the weld pool, leading to a decrease in weld penetration.

### 3.4. Optimal Parameter Window for the Welding Process

**Figure 13:**
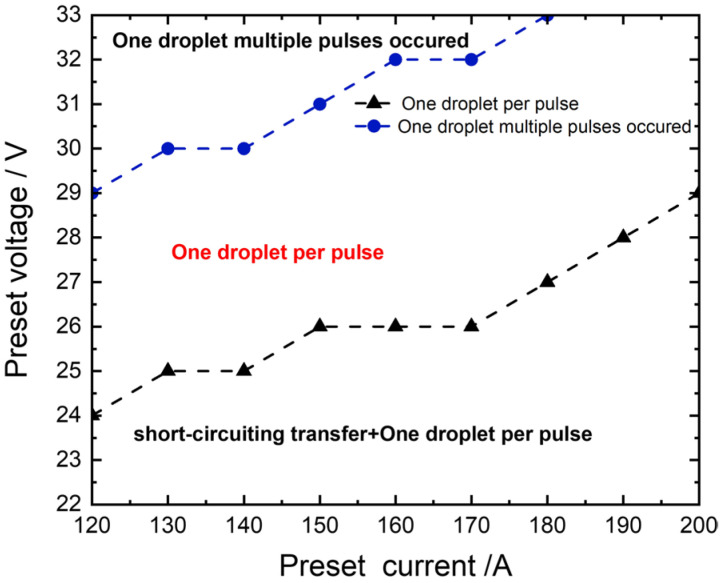
Optimal parameter window for the welding process.

The metal transfer and arc behavior, particularly for solid wires, were significantly influenced by both the preset current and voltage, thereby subsequently impacting the weld formation. The optimal parameter window for the welding process was obtained based on the analysis above, as illustrated in Figure 13. The flux-cored wire maintained multiple droplets per pulse due to its easily meltable properties. When the preset voltage was lower, i.e., the shorter arc, the droplets at wire ends were prone to contacting the weld pool to form a short-circuiting transfer, and the frequency of short-circuiting transfer decreased with increasing preset voltage. Furthermore, short-circuiting transfer would lead to poorer welding stability, thereby affecting the final weld formation. The metal transfer mode transitioned into one droplet per pulse with an increase in preset voltage, which was the desired metal transfer mode. When the preset voltage gradually increased until it reached a certain threshold, the one droplet multiple pulses started to occur in the welding process, and interference among arcs became more intense. Consequently, abnormal arc ignition frequently occurred, resulting in decreased welding stability and weld offset. In addition, when the preset current was higher, it also led to short-circuiting transfer but with a faster frequency of metal transfer. In conclusion, the optimal parameter window was identified as matching the electrical parameters corresponding to one droplet per pulse. The welding process could achieve excellent welding stability and produce better weld formations within this parameter window.

In addition, within this parameter window, a heat input of 13.65 kJ/min was selected for the welding process. The post-weld deposition rate was evaluated for triple-wire, twin-wire, and single-wire welding processes, respectively. Experimental results indicated that the deposition rate achieved after simultaneous arcing of triple-wires was 7.27 kg/h, while it was 5.26 kg/h for twin-wire welding and 3.53 kg/h for single-wire welding. This application of triple-wire welding for UHSS demonstrated a significant enhancement in post-weld deposition rate, with an increase of 106% and 38% compared to single-wire and twin-wire welding techniques, respectively. A significant improvement in welding efficiency could be achieved through the simultaneous arcing of triple-wire during welding.

### 3.5. Analysis of Joint Microstructure and Mechanical Properties

Based on the above welding parameter window, the optimized parameters, i.e., a preset voltage of 26 V and a preset current of 140 A, were selected for butt welding to evaluate the mechanical performance of the welded joint. Additionally, the research was conducted on the microstructure, microhardness, and mechanical properties of the welded joint under these appropriately matched electrical parameters.

#### Analysis of Microstructure Characteristics

**Figure 14:**
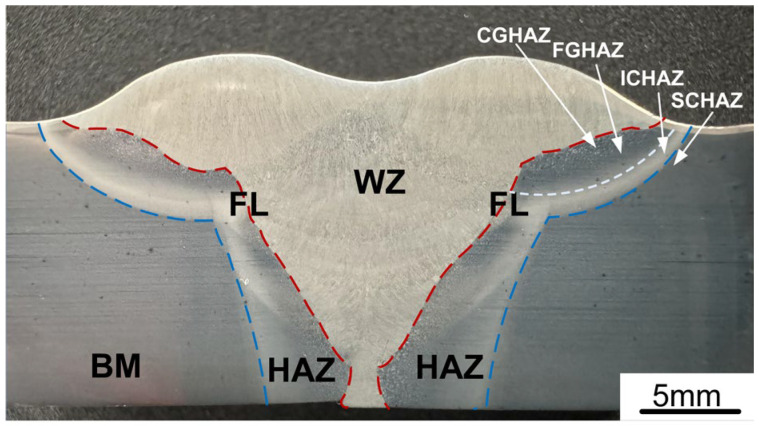
Cross-section profile of the welded joint.

The cross-sectional profile of the joint at 140 A and 26 V is illustrated in Figure 14. The welded joint was fully penetrated without any macroscopic defects such as cracks, incomplete fusion, or porosity. The welded joint can be divided into three regions: the weld zone (WZ), the heat-affected zone (HAZ), and the base metal (BM). Additionally, the HAZ can be further divided into sub-zones: the coarse-grained HAZ (CGHAZ) closest to the fusion line (FL), the fine-grained HAZ (FGHAZ), the intercritical HAZ (ICHAZ), and the sub-critical HAZ (SCHAZ). Among these sub-zones, the ICHAZ experienced the highest peak temperature, which caused an increased grain size in this region.

**Figure 15:**
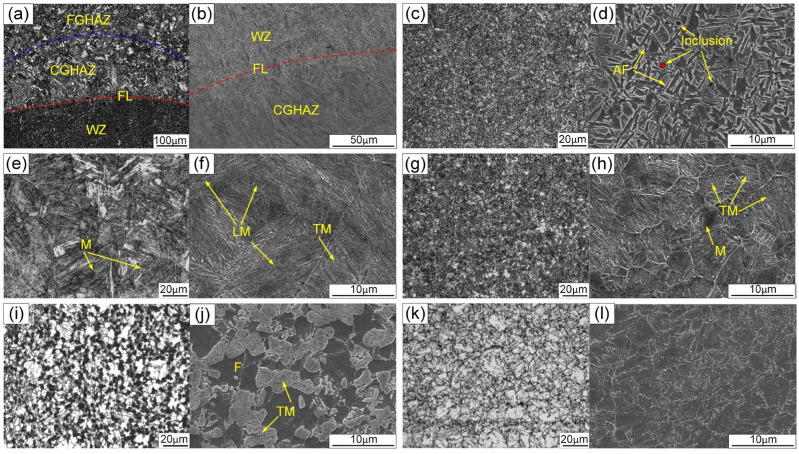
Microstructures observed via OM and SEM in different sub-zones for the upper part of the joint: (**a**,**b**) evolution within each subzone, (**c**,**d**) weld zone (WZ), (**e**,**f**) coarse-grained HAZ (CGHAZ), (**g**,**h**) fine-grained HAZ (FGHAZ), (**i**,**j**) intercritical HAZ (ICHAZ), and (**k**,**l**) subcritical HAZ (SCHAZ).

**Figure 16:**
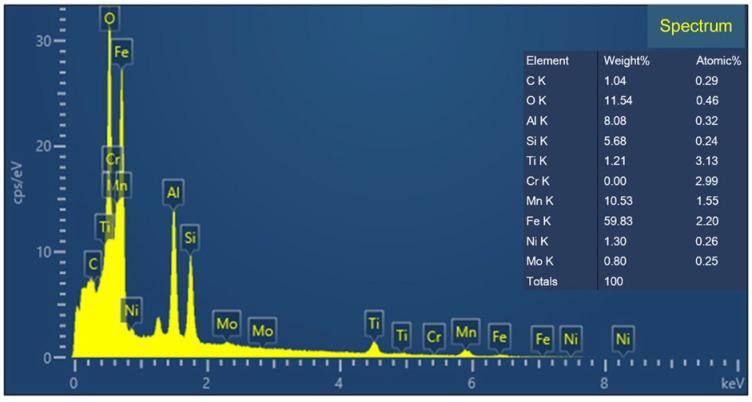
Spectrum of inclusion nucleating acicular ferrite.

The microstructures of each distinct subzone observed using OM and SEM for the upper part of the welded joint are depicted in Figure 15. In the WZ, several nonmetallic inclusions, such as oxides, could serve as nucleation sites for acicular ferrite (AF). The chemical elements of inclusion marked with a red cross in Figure 15d were identified using energy-dispersive X-ray spectroscopy (EDS). As presented in Figure 16, the EDS analysis indicated that the inclusions were rich in Al, O, Si, Mn, etc. Al easily reacts with O to form Al_2_O_3_ microinclusions, which are conducive to AF nucleation in the weld metal. This was because the high-energy inert interfaces of the inclusions can effectively reduce the potential barrier for AF nucleation [31]. The expansion coefficients of inclusions and the weld metal matrix are different, thereby facilitating the formation of a high-stress zone that promotes AF nucleation [23]. As a result, AF predominated in the WZ. Due to its refined and interwoven structure, AF could provide an optimal combination of high strength and good toughness. The microstructure of the CGHAZ was a mixture of lath martensite (LM) characterized by a lath structure and tempered martensite (TM), both with larger grain sizes [32]. The microstructure of the FGHAZ mainly consisted of fine-grained TM mixed with some M [33,34]. The ICHAZ experienced welding thermal cycles between A_c1_ and A_c3_. Consequently, the partially transformed austenite transferred into TM after cooling, while the untransformed ferrite (F) only underwent heating and growth during the welding thermal cycles [35]. The SCHAZ exhibited a microstructure similar to that of the BM.

### 3.6. Analysis of Joint Mechanical Properties

#### 3.6.1. Microhardness

**Figure 17:**
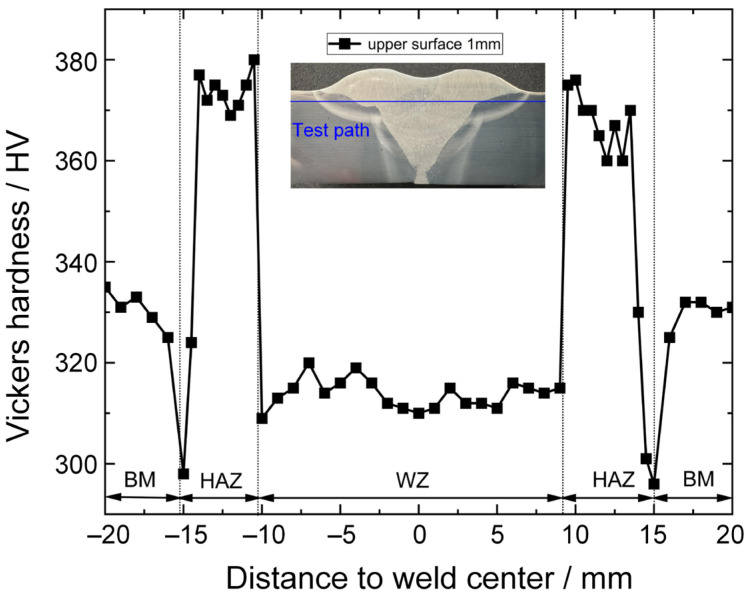
Microhardness distribution characteristics of the joint.

The experiment employed the microhardness tester model TDMH-3 for measurements. The hardness values were measured at intervals of 1 mm along the weld zone and 0.5 mm along the HAZ and the BM. A load of 1 kgf was applied for 15 s during testing. It can be observed that the joint microhardness exhibited a generally symmetrical distribution on both sides of the weld zone, as illustrated in Figure 17. The WZ in the welded joint displayed the lowest and relatively stable microhardness, measuring approximately 314 HV. However, greater variations were observed in the microhardness of the whole HAZ. Specifically, the microhardness of the HAZ adjacent to the WZ sharply increased to a maximum value of 380 HV, while it suddenly decreased to a minimum value of 296 HV closer to the BM. This variation was mainly because the HAZ adjacent to the WZ consisted of coarse grains, particularly the coarse M. As a result, the microhardness increased to the maximum value. Nevertheless, the HAZ adjacent to the base metal was an incomplete quenching region composed of TM and F; therefore, it exhibited the lowest microhardness. Additionally, the microhardness of the BM remained relatively constant at 332 HV.

#### 3.6.2. Tensile Property

**Figure 18:**
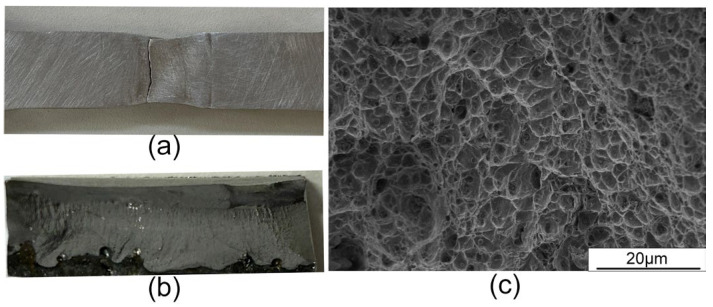
Fracture location and macro- and microfracture morphologies of the tensile specimens: (**a**) fracture location; (**b**) macroscopic fracture; (**c**) microscopic fracture.

The average tensile strength of the joint reached 928 MPa, which was approximately 88% of the base material strength (1060 MPa). The average elongation and reduction in the area were measured at 12.6% and 23.2%, respectively. Figure 18 illustrates the fracture location and morphology of the joint, where all specimens fractured in the WZ with clear necking observed at the fracture location, as depicted in Figure 18a. The overall morphology of the fractured surface is described in Figure 18b. The microfracture surface exhibited distinct dimples, and various-sized inclusions could be found at the bottom of the dimple, as depicted in Figure 18c.

#### 3.6.3. Low-Temperature Impact Toughness

The V-notches were machined in the WZ and HAZ, respectively, and three specimens were selected from each of the above two areas to study the low-temperature impact toughness of the joint at −40 °C. The average impact absorbed energy of WZ and HAZ could reach 54 J and 126 J, respectively. Despite the higher heat input due to three arcs burning simultaneously, the joint still exhibited excellent low-temperature impact toughness.

**Figure 19:**
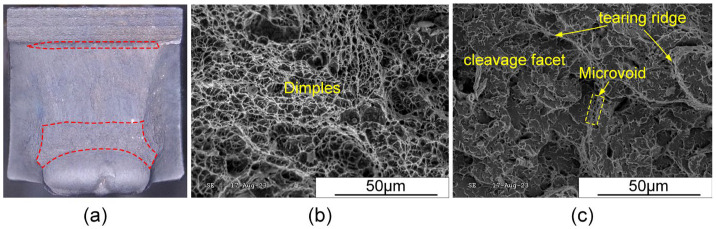
Macroscopic and microscopic-fractured surfaces of WZ after Charpy impact testing at −40 °C: (**a**) macroscopic fracture, (**b**) ductile fracture region, and (**c**) quasi-cleavage fracture region.

**Figure 20:**
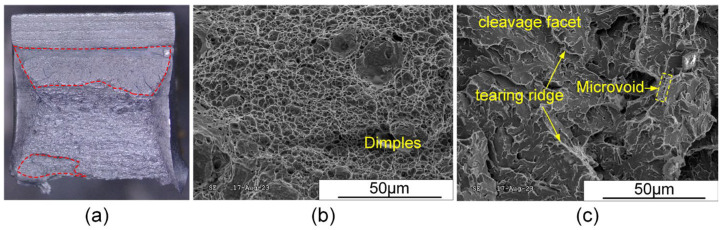
Macroscopic and microscopic-fractured surfaces of HAZ after Charpy impact testing at −40 °C: (**a**) macroscopic fracture, (**b**) ductile fracture region, and (**c**) quasi-cleavage fracture region.

Figure 19 and Figure 20 illustrate the morphologies of the impact fracture surfaces for the WM and HAZ, respectively. In the following figures, the red region represents the fibrous zone, and the larger the area of the fiber region, the better the joint toughness. It can be observed that the HAZ exhibited a larger fibrous zone, resulting in higher impact absorbed energy compared to the WZ, as depicted in Figure 19a and Figure 20a. The fracture morphologies of the WM and HAZ were mixed fractures consisting of both quasi-cleavage and ductile fractures. The ductile fracture region exhibited tearing ridges with many fine dimples, as depicted in Figure 19b and Figure 20b, while the quasi-cleavage fracture region consisted of flat cleavage facet, microvoids, and tearing edges, as shown in Figure 19c and Figure 20c. Furthermore, the ductile fracture was characterized by microvoid coalescence, which contributed to increased crack propagation absorbed energy.

## 4. Conclusions

In this paper, a novel type of welding process for UHSS is proposed to achieve high efficiency and quality. Furthermore, extensive research is conducted to reveal the effects of electrical parameters on metal transfer, arc behavior, and weld formation to obtain the optimal parameters window. In addition, the joint microstructure and mechanical properties were analyzed. The following conclusions can be drawn from this study:

(1) The metal transfer modes of solid wires were mainly determined by welding electrical parameters. In contrast, the flux-cored wire consistently maintained multiple droplets per pulse due to the longer duration of the pulse peak phase. When the preset voltage was lower or the preset current was higher, the solid wire easily generated short-circuiting transfer, which not only decreased the welding stability but also caused a “sawtooth-shaped” weld formation. Furthermore, when the preset voltage increased to a certain value, the solid wire would generate the metal transfer of one droplet multiple pulses, and abnormal arc ignition frequently occurred. This led to decreased welding stability and weld offset towards one side with more spatters. The electrical parameters corresponding to one droplet per pulse were identified as the optimal parameter window.
(2) The presence of oxides in the WZ facilitated the nucleation of AF; thus, the WZ was predominantly composed of AF, which offered an optimal combination of high strength and good toughness. The microstructure of the CGHAZ consisted of LM and TM with larger grain sizes, leading to increased microhardness up to a maximum value of 380 HV. The microstructure of the FGHAZ mainly consisted of fine-grained TM mixed with some M. The ICHAZ underwent incomplete phase transformation; as a result, the microstructure consisted of untransformed F and TM, which exhibited the lowest microhardness of 296 HV.
(3) Despite the higher welding heat input, the welded joint still showed excellent mechanical properties, especially toughness. The average tensile strength of the joint could reach 928 MPa, which was approximately 88% of the base material strength. Additionally, the welded joint exhibited improved impact toughness at low temperatures of −40 °C, with impact absorbed energy values of both WZ and HAZ reaching 54 J and 126 J, respectively. This was because the microstructures of WZ and HAZ were composed of AF and TM, respectively, both of which exhibited excellent strength and toughness.
(4) A medium-thickness plate of UHSS was primarily welded using a single electrode; as a result, welding efficiency remained somewhat limited. Significant improvement in welding efficiency could be achieved through the simultaneous arcing of three wires. When the welding heat input was kept constant, the application of triple-wire welding for UHSS demonstrated a significant enhancement in post-weld deposition rate, with an increase of 106% and 38% compared to single-wire and twin-wire welding techniques, respectively.

## Figures and Tables

**Table 1:** Chemical composition of welding materials (wt%).

No.	C	Mn	Si	S	P	Ni	Mo	Cr	Ti	Cu	V	Al	Nb
Q960E	0.15	1.11	0.29	0.0018	0.007	0.31	0.55	0.20	0.022	0.04	0.049	0.049	0.02
JQ·MG90-G	≤0.11	1.6~1.9	0.4~0.8	≤0.025	≤0.025	2~2.5	0.5~0.8	0.2~0.6	≤0.12	≤0.5	-	-	-
JQ·YJ80ML	0.063	1.72	0.47	0.004	0.007	2.58	0.49	0.021	0.024	-	-	-	-

**Table 2:** Mechanical properties of Q960E steel.

Tensile Strength (MPa)	Yield Strength (MPa)	Elongation (%)	Impact Toughness at −40 °C (J)
1060	1032	15	71

## Data Availability

The original contributions presented in the study are included in the article, further inquiries can be directed to the corresponding author.
